# The Relation among Prostate Cancer Knowledge and Psychosocial Factors for Prostate Cancer Screening among African American Men: a Correlational Study

**DOI:** 10.3934/publichealth.2017.5.446

**Published:** 2017-10-25

**Authors:** Sabrina L. Dickey, Aurellia Whitmore, Ellen Campbell

**Affiliations:** 1 Florida State University, College of Nursing, Tallahassee, Florida, USA; 2 Florida Agricultural & Mechanical University, Tallahassee, Florida, USA

**Keywords:** African American men, prostate cancer, prostate cancer screening, prostate cancer knowledge

## Abstract

African American (AA) men face disproportionately higher rates of prostate cancer (PCa) in comparison to other races. In addition, higher mortality rates from PCa amongst AA men signifies PCa as a formidable health disparity. Inconsistent PCa screening guidelines among medical organizations, further clouds one's decision on receiving a PCa screening. Examining various relations among factors which influence PCa screening may provide insight into their decision whether or not to receive a PCa screening. The purpose of the study was to examine the presence of associations among PCa knowledge, psychosocial factors, and PCa screening over a six month time frame. There were 76 participants at baseline, intervention group (n = 37) and control group (n = 35) and 54 participants, intervention group (n = 26) and control group (n = 28) remained at the 6 month follow up. At the six month follow up, the control group was more likely to have not received a PCa screening and the intervention group was more likely to have received a PCa screening, *p* < 0.01. PCa knowledge scores rose from 49% to 71%, intervention group, and 52% to 58%, control group. Significant associations were found among the following covariates, age and religion (*rs* = 0.499, *p* < 0.01), income and education (*rs* = 0.535, *p* < 0.01), income and healthcare coverage (*rs* = 0.528, *p* < 0.01), income and PCa knowledge at 6 months (*rs* = 0.424, *p* < 0.01), PCa screening and religion (*rs* = 0.353, *p* < 0.01), healthcare empowerment and preparation for decision making (*rs* = 0.421, *p* < 0.01), decisional self-efficacy and active surveillance knowledge (*rs* = 0.377, *p* < 0.01), and active surveillance knowledge and PCa knowledge (*rs* = 0.497, *p* < 0.01). The study revealed associations among PCa knowledge and psychosocial factors regarding a decision for PCa screening among the PCa high risk group, AA men.

## 1. Introduction

Prostate cancer (PCa) is the second most common cancer in the United States (U.S.). In 2016, there were an estimated 180,890 new cases of PCa [1]. Studies have shown that African American (AA) men have a genetic tendency to be diagnosed with PCa [2]. It is well known that race, African American or Black, is a risk factor for developing PCa. Mortality rates for Black men with PCa are more than twice those of White men [3]. Prostate cancer is known to progress slowly, and its five-year survival rate is 98.9% [1]. It is important to note that despite the high five-year survival rate, AA men still experience higher incidence and mortality rates, as well as increased adverse outcomes from PCa [1]–[3].

### 1.1. Prostate cancer screening

The digital rectal exam (DRE) and the protein-specific antigen (PSA) test are first-line screening measures for PCa. Since the introduction of the PSA test in the 1980s, the DRE has been used to a lesser extent. The PSA test measures the level of PSA in the blood, which is produced by the prostate gland. In contrast, the DRE is an exam that evaluates the anatomy of the prostate gland through the male's rectum. According to the American Cancer Society (ACS), (2016) the DRE is less effective at finding PCa than the PSA test [3]. Despite this situation, the DRE has been shown to identify PCa in men whose PSA blood levels are normal [3]. In actuality, the DRE and PSA have been the mainstays for PCa screening in the United States for the last 25 years [4]. The combined use of the DRE and the PSA test was proven to be the most beneficial for detection of PCa within three uncontrolled studies [5]. Previous literature also advocated for the combined use of the PSA test and the DRE for PCa screening [6]. Prostate cancer screening in itself is associated with psychological stigmas among many men [7]. The stigmas vary from embarrassment and discomfort with receiving the DRE to the possibility of erectile dysfunction if diagnosed and surgically treated for PCa [7]. Lee, Consedine, and Spencer (2011) noted that AA men had significantly more fear of PCa screening than White men [8]. The fears associated with PCa screening may explain why AA men have fairly low rates for screening [9].

There is no single predictor of AA men's decision to receive PCa screening. Instead, the decision is multidimensional and involves psychosocial and psychological factors. Access to healthcare and demographic factors have served as barriers and facilitators for receiving PCa screening [10],[11]. Education is a pertinent factor for increasing awareness and identifying the risk factors for PCa among AA men.

There is no doubt that inconsistencies exist in surrounding guidelines for PCa screening. In 2012, the United States Preventive Services Task Force (USPSTF) established the precedence for recommending against PCa screening and providing a recommendation grade of D. In essence, the USPSTF stated it is more harmful to receive PCa screening and that it confers no benefit to the life expectancy of an individual [12]. It is important to note that the USPSTF's recommendation was determined from the results of the two largest PCa screening trials, the European Randomized Study of Screening for Prostate Cancer and the Prostate, Lung, Colorectal and Ovarian Cancer Screening Trial (PLCO), which both severely lacked racial diversity. In contrast to the USPSTF, the ACS recommends that men discuss the risks and benefits of PCa screening with their healthcare provider prior to making a decision about screening [3]. Additionally, the American Urological Association stipulates that men between the ages of 40 and 54 years of age, at average risk for PCa, should not be routinely screened for PCa [13]. The USPSTF issued a draft recommendation statement for PCa screening in 2017 that changed from a grade D to that of a C [14]. The USPSTF changed its stance from recommending against PCa screening to that of PCa screening be an individual decision for men ages 55–69. The draft advised clinicians to inform men on the potential harms and benefits of PCa screening using the PSA test, which will allow for individualized decision making for PCa screening [14]. Overall, the lack of consistent guidelines does not provide direction for the highest at risk group for developing PCa, AA men.

### 1.2. Religiosity

Religion is used interchangeably with the term religiosity and is defined as institutional with boundaries and encompassing social properties, which makes it different from that of spirituality [15]. Furthermore, religiosity is comprised of a system of formal beliefs, belief in a higher power, and service attendance [16]. Religion as a whole, is viewed as a vital source for influencing changes to behavior and health in general [15],[17]. Empirical studies indicated there are three primary modes that religion influences health (1) Stress reduction; (2) Social support; and (3) encouraging healthier living habits [18]. Leyva et al. (2014) [16] examined the relationship between religiosity and cancer screening and found a strong relation between attending religious activities and early cancer screenings. The prescribed beliefs and traditions of religiosity to live a life that is pleasing to a higher power may invoke a desire to engage in health promoting activities. Furthermore, a desire to live a healthier life can provide the incentive to make a decision for health screening, which can impact an individual's self-efficacy.

### 1.3. Self- efficacy and decision making

The theory of self-efficacy involves the ideation of an individual's ability to engage in a particular behavior [19]. Bandura (1977) identified self-efficacy as a central concept for identifying one's cognitive ability to complete a task or change a behavior despite the context of a situation [20],[21]. When measuring self-efficacy, it is linked to fulfillment of a specific task [19]. Self-efficacy is a vital concept to the empowerment of an individual to make a good decision. Therefore, self-efficacy and decision-making are often examined together when information is provided and an individual must make a choice. In order to promote self-efficacy, which in turn can lead one to make a decision, individuals must feel encouraged and possess a belief that they are capable of achieving a particular goal [22]. In essence, an individual's sense of efficacy is linked to his/her belief in the ability to produce a change in behavior [23]. Ultimately, a change in behavior will occur when individuals are motivated by a positive outcome [23]. Through providing information on the health behavior of PCa screening, a foundation for developing or reigniting a sense of self-efficacy for completing a PCa screening can be formulated. Once the information is provided the next step will involve the role of decision making for receiving a PCa screening.

In order for patients to make good decisions, they must be knowledgeable regarding a particular topic [24]. Physicians often have limited time to pursue in-depth discussions with their patients regarding their concerns and fears surrounding screening [25]. The difficulty of making a decision is exacerbated among populations, which lack financial resources and access to healthcare. The importance of decision making has gained a spotlight in healthcare as a result of various factors in today's society (1) increased in patient autonomy; (2) increased access to information; (3) expanded clinical options; (4) increased costs; (5) increased chronic illnesses [24]. Literature, affirms that when AA men are exposed to information about the importance of screening, whether it is from their spouse, peers, community-based educational programs or mass media, they are more likely to engage in discussions with their health care providers about PCa screening [26]. Furthermore, they are more likely to engage in discussions regarding treatment options should they test positive, and subsequently make informed decisions related to diagnosis and management [26]–[28]. Prior to making a decision regarding PCa, it is imperative that individuals are knowledgeable regarding the risks and benefits of the screening [29].

Local sites within the AA community, such as barbershops and churches, can be vital venues for the dissemination of health information [30]. Additionally, there is extensive literature regarding the possibility of urinary incontinence and erectile dysfunction from the medical treatments for PCa [31]. Unfortunately, the possibility of negative consequences from PCa treatment may impede men from discussing what they consider to be a sensitive topic [31],[32]. Despite some men identifying the potential of urinary incontinence and erectile dysfunction as an uncomfortable topic, it should be incorporated into education regarding PCa screening. In doing so, men will have an opportunity for a well-rounded education of PCa and PCa screening, which includes the benefits and risks. A focus on providing health information is a vital concept within the AA population, which is known for having decreased rates of engaging in preventative health behaviors [33]. The transmission of health information is further compounded among AA individuals due to their generational history of maintaining secrecy surrounding illnesses within the family [34]. Therefore, it is essential to overcome communication barriers and provide educational sessions for AA men on health-promoting behaviors within their communities. An exploration of one's level of educational awareness of PCa and one's level of self-efficacy for making a decision to receive PCa screening can provide insights into the motivators for AA men to receive PCa screening. The purpose of the study was to examine the existence of relations among PCa knowledge and screening, active surveillance (AS) knowledge and psychosocial factors (demographics, religion, decisional self-efficacy, preparation for making decisions, and healthcare empowerment for making healthcare decisions) among AA men in a six-month follow up educational study. The identification of relations among the covariates after a six-month follow up provides valuable data regarding the associations of factors and AA men's decision making for health promotion and screening behaviors such as PCa.

## 2. Materials and methods

### 2.1. Design, setting, sample

A quasi-experimental design with an educational intervention was implemented for the study. Pre- and post-tests were utilized to evaluate relations among the covariates for AA men after a six-month follow up. A city in northeastern Florida provided the setting for the study. We focused on 40-year-old and over, English-speaking AA men who had never received PCa screening or whose last screening had occurred over a year ago, and had never been diagnosed with PCa. Due to the prevalence of PCa among AA men we sought to include participants which were at least 40 years of age, which will increase their exposure to PCa and screening. Local churches, masonic organizations, fraternal organizations, and philanthropic organizations within the AA community were recruited and designated as either an intervention or a control group. Only the intervention groups were provided with educational interventions for PCa and PCa screening. Funding was not available to provide free PCa screening, therefore participants were provided with information regarding medical facilities and institutions that provided PCa screening. To increase the comfort level of the men and to encourage health discussions among families, the spouses and/or partners of AA men were invited to attend the educational intervention. Churches or community centers served as the venue for completing the baseline surveys. Upon establishing eligibility to participate in the study, baseline questionnaires were administered to the participants in both groups. Participants in the intervention arm of the study first completed the pre-tests. Immediately following the pre-tests, they viewed a video, the educational intervention. The video was obtained from the U.S. National Library of Medicine, National Institutes of Health, National Cancer Institute. The video appealed to the lay person in that it was succinct, a short length of approximately two minutes, and very easy to understand. The contents of the video contained information regarding cancer etiology, anatomy and physiology of the prostate, PCa screening, diagnosis of PCa, and treatment and preventive behaviors. After the video was shown to the education groups a question and answer session regarding the information discussed in the video was initiated by the primary investigator. The post-tests were administered after the video. Individuals in the control groups did not receive the educational intervention and only completed the baseline questionnaires. A $25 gift card was provided to the participants in the intervention and control groups upon completing the baseline pre- and post-tests.

Six months after each intervention and control group completed the baseline questionnaires, the participants were contacted via phone for follow up. During the phone call, information about the participant's last PCa screening was obtained. Additionally, a new set of responses from the baseline questionnaires was obtained from the participants via the phone call. Participants were not provided with their previous responses during the follow up phone call. A $10 gift card was distributed as an incentive for retention, via certified mail, to the participants who completed the six-month follow up.

### 2.2. Measures

Each participant's demographic data were obtained utilizing the Behavioral Risk Factor Surveillance System (BRFSS). The Center for Disease Control and Prevention (CDC) has used this survey to obtain health risk data pertaining to the leading causes of morbidity and mortality by phone across the nation since its inception in 1984. The BRFSS collects data on an annual basis. The survey contains core items developed by the CDC, as well as optional modules and state-added questions. The current study included sections from the 2004 and 2013 BRFSS surveys that contained questions pertaining to PCa screening and status. Demographic questions, which were unaltered, from the BRFSS questionnaire pertaining to age, education, income, and marital status was utilized to collect the demographic data of the participants.

### 2.3. PCa screening status

The PCa screening status of participants was examined using three questions on the BRFSS. Two questions consisted of the last DRE and PSA screening, and the responses were rated as 1 (less than 1 year), 2 (more than 1 years but fewer than 2 years), 3 (more than 2 years but fewer than 3 years), 4 (more than 4 years but fewer than 5 years), 5 (5 or more years), 6 (don't know; not sure). The final question asked whether participants had received PCa screening and was rated as 0 (no), 1 (yes), or 2 (don't know; not sure). At the six-month follow up, PCa screening status was determined by the question, “What was the date of your last PCa screening?” Responses were categorized as 0 (never), 1 (within 1 year), 2 (more than 1 year ago but fewer than 2 years), and 3 (more than 2 years ago).

*PCa knowledge scale:* The PCa knowledge questionnaire consists of 10 items, and it evaluates a participant's knowledge of PCa [35]. The scale utilized in the current study included 4 additional items which were examined by Partin et al., (2004) and from previous studies [35]. In all, there were a total of 14 questions that assessed PCa knowledge. Responses were rated as “true”, “false” or “I don't know”, and a point value was assigned to each correct answer. The total score ranges from 0 (no correct answers) to 100 (all correct answers). A higher score is associated with increased PCa knowledge [36]. There was high internal reliability in studies regarding informed decision making for PCa among AA men (Cronbach's α = 0.79) [35],[37]. Despite the scale's existence since 2004, the answers were verified to determine accuracy of what is currently known regarding PCa and the information was still accurate. A comparison of questions on the scale to current information on the three sections of the scale, risk factors for PCa, follow-up tests, and treatment complications was conducted without any erroneous information being found.

### 2.4. Religiosity

The Religiosity Scale, developed by Lukwago, Kreuter, Buchotz, Holt, and Clark (2001), measures participants' level of religiosity [38]. The scale consists of nine items. Previous studies regarding PCa screening and urban AA women have utilized the scale to assess religiosity among participants [38],[39]. Responses for the scale were rated as 1 (strongly disagree), 2 (disagree), 3 (agree), and 4 (strongly agree). A high degree of religiosity is indicated by a higher score. The literature reports a suitable Cronbach's α of 0.88 [38],[39].

### 2.5. Decision self-efficacy scale and active surveillance

The decision self-efficacy scale (DSES) evaluates self-confidence in making decisions, belief in one's ability to make a decision, and shared decision-making capacity [40]. The scale consists of 11 items, and responses are on a five-point scale ranging from “not at all confident” to “very confident”. A higher score is indicative of increased self-confidence in making decisions. The DSES has demonstrated high internal reliability (Cronbach's α = 0.92) in previous studies [37],[41]. Knowledge of AS was evaluated by six questions from the ACS. Participants selected one of the following responses: 0 (no, false), 1 (yes, true), or 2 (don't know; not sure).

Preparation for Decision Making Scale (PDMS) and HealthCare Empowerment Questionnaire (HCEQ)

To assess the impact of the study on the participant's preparation for decision making about PCa screening, the Preparation for Decision Making Scale (PDMS) was administered [42]. In a study of informed decision making (IDM) among church-attending AA, the PDMS has been shown to have high internal reliability (Cronbach's α = 0.99) [40]. The scale consists of 10 items using a five-point Likert scale with responses ranging from 1 (“not at all”) to 5 (“a great deal”). Scores were summed and converted to a 0–100 scale with higher scores indicating a higher perceived level of preparation for decision making. A test-test reliability score of 0.94 has been reported [42].

The HCEQ assesses a participant's sense of empowerment for seeking healthcare and his or her own personal health. The scale consists of 10 questions associated with seeking care and 10 questions pertaining to the importance of health information. Each set of 10 questions has a four-point Likert scale: 1 (not at all), 2 (somewhat), 3 (very much), 4 (extremely). Scores range from 1–16, and higher scores indicate a higher degree of personal healthcare empowerment. A Cronbach's α of 0.83 has been reported for this scale [43].

### 2.6. Statistical analysis

We used the IBM Statistical Package for the Social Sciences (SPSS) version 22 (IBM Corp., Armonk, NY) for the descriptive and statistical analysis. The descriptive statistics consisted of means, standard deviations, frequencies, and percentages. Demographic data displayed each participant's personal characteristics as well as his or her social economical status. An independent samples *t*-test evaluated differences among the experimental and control groups regarding the covariates addressed in the study. Cross tabulations compared the frequency and percentages for PCa knowledge level among the intervention and control groups. Chi-squared statistics examined the significance of PCa screening. Spearman's Rho correlations were implemented to establish the presence and strength of an association among the covariates. The level of significance was set at *p* < 0.05 for the statistical data.

## 3. Results

At the onset of the study, 76 AA men consented to participate in either the experimental (*n* = 37, 48.7%) or control (*n* = 39, 51.3%) groups. Most of the participants were married (*n* = 31, 58.5%), had completed high school or obtained a GED, and had attended college for 1–3 years. had the same results (*n* = 18, 33.3%) and an income of $50,000 and over (*n* = 25, 48.1%). Table 1 lists the demographic data of the cohort.

By the six-month follow up, the total sample size included 54 individuals, listed as follows; in the experimental (*n* = 26, 48.1%) and control (*n* = 28, 51.9%) groups. The loss of participants yielded an attrition rate of 29%. At the six-month follow up, a total of 22 men did not participate due to non-working phone numbers and/or an indication of a lack of desire to participate in the study.

A participant's decision to receive PCa screening at the six-month follow up was examined using the Pearson's chi-squared test. Significant results were obtained regarding the screening status of participants in the intervention and control groups (*p* < 0.05). At the conclusion of the study, 14 participants had received PCa screening. Cross tabulations and a chi-squared analysis for the overall PCa screening results for the intervention and control groups indicating “yes” or “no” to receiving a PCa screening are reported in Table 2.

**Table 1.** Descriptive statistics (n = 54).

*Variable*	*n or %*	*M*
*Prostate Cancer Screening*		
Baseline		
Experimental group		
Never	11	
>1 year or ≥2 years	18	
= or >3 years	3	
Control group		
Never	21	
>1 year or ≥2 years	12	
= or >3 years	2	

*Controls*		
*Age*		51
*Education*		
Grades 9–11	6%	
Grade 12 or GED	35.8%	
College 1 to 3 years or technical school	32.8%	
College 4 years or more and graduate	25.4%	
School		
*Income*		
Under less than $10, 000	3%	
$10,000 to less than $15,000	4.5%	
$15,000 to less than $20,000	11.9%	
$20,000 to less than $25,000	11.9%	
$25,000 to less than $35,000	11.9%	
$35,000 to less than $50,000	6%	
$50,000 and over	47.8%	
Don't know not sure	3%	
*Marital* status		
Married	56.1%	
Divorced	19.4%	
Widowed	1.5%	
Separated	4.5%	
Never married	14.9%	
Member of unmarried couple	3%	

**Note**: *n* = 54; **Abbreviations:** *M* = Mean; % = percentage; SD, standard deviation; Min, Minimum; Max, Maximum.

**Table 2.** Overall Crosstab for the 6 month follow up prostate cancer screening.

	Control	Experimental	Total
*Yes*	3 (21.4%)	11 (78.6%)	14
*No*	25 (62.5%)	15 (37.5%)	40
Chi square 7.0 *			

* *p* < 0.05.

The intervention group was significant for not receiving PCa screening at the six-month follow up (*n* = 15, 37.5%). A critical cut-off limit for the results of ±1.96 was established among the groups for the responses of “no” or “yes” for receiving PCa screening at the six-month follow up. An adjusted residual of −2.6 for the intervention group signified that these participants were less likely to have not received PCa screening compared with their control-group counterparts. The control group was also significant and had a higher rate for “no” regarding PCa screening (*n* = 25, 62.5%). In essence, participants in the control group were more likely to have not received PCa screening at the six-month follow up based on adjusted residuals of 2.6. There were also significant results among the intervention and control-group participants who responded “yes” to receiving PCa screening at the six-month follow up. The intervention group was deemed to be more likely to be screened at the six-month follow up based on the positive adjusted residuals (2.6). Eleven participants in the intervention group responded “yes” for receiving PCa screening at the six-month follow up (76.6%). Three individuals from the control group also responded “yes” (21.4%). Participants in the control group were less likely to receive PCa screening at the six-month follow up as evidenced by a negative adjusted residual (−2.6). One of the inclusion criteria required for participation in the study was that participants must had never been screened for PCa or their last previous screening must have occurred over one year ago. Therefore, additional analysis was conducted to determine the length of time between a participant's last PCa screening and his six-month follow up phone call. Table 3 lists the cross tabulations and Pearson's chi-squared results for the length of time between a participant's last PCa screening at the time of the six-month follow up and their baseline response of never having received PCa screening or PCa screening occurred over one year ago. The category of less than or equal to one year was included to reflect participants who received a PCa screening during the six month follow up time frame. Results from Table 3 revealed a total of 14 men received a PCa screening after participation in the study as evidenced by *n* = 3 (21%) for the control group and *n* = 11 (78.6%) for the experimental group having received a PCa screening in less than or equal to 1 year since their last PCa screening.

**Table 3.** Crosstab and Chi square for the length of time between last prostate cancer screening at the six month follow up.

	*Control*	*Experimental*	*Total*
*Never received a prostate cancer screening*	12 (85.7%)	2 (14.3%)	14
*< or = 1 year*	3 (21.4. %)	11 (78.6%)	14
*>1 year or =2 years*	9 (56.3%)	7 (43.8%)	16
*>2 years*	4 (40%)	6 (60%)	10
Chi square 12.3 *			

* *p* < 0.05.

Pearson's chi-squared analysis for the length of time since a participant's last PCa screening at the six-month follow up yielded significant results (*x^2^* = 12.30, *p* < 0.01). The PCa screening rates of “never”, “greater than or equal to one year”, “greater than one year or equal to 2 years” and “greater than two years” were compared between individuals in the intervention and control groups. Adjusted residuals across each group were obtained to assess the significance of each cell. A critical cut-off limit for the significance of the cells was set at ±1.96. There were two categories that met the critical cut-off limit: “never” and “less than or equal to one year.” The intervention group was found to be less likely to have never received PCa screening at the six-month follow up (*n* = 2, 14.3%) for an adjusted residual of −2.9. Individuals in the control group were more likely to have never received PCa screening at the six-month follow up (*n* = 12, 85.7%) for an adjusted residual of 2.9. The results for receiving PCa screening at less than or equal to one year at the six -month follow up for the intervention group were *n* = 11 (78.6%) for an adjusted residual of 2.6. Therefore, the intervention group was more likely to have received PCa screening within one year of their last reported PCa screening. In contrast, individuals in the control group were less likely to have received PCa screening within one year: *n* = 3 (21.4%) for an adjusted residual of −2.6.

We also conducted data analysis for PCa knowledge. The PCa knowledge score at the baseline for both groups indicated a correct response rate with a mean of 51%. By the six-month follow up, the mean of the correct response rate for PCa knowledge had increased to 64%. A comparison of PCa knowledge among the intervention and control groups indicated an increase in both groups. The baseline PCa knowledge correct mean score for the intervention group was 49% compared with 71% at the six-month follow up. Results for the baseline PCa knowledge mean correct scores for the control group was 52% compared with 58% at the six-month follow up. Furthermore, an independent samples *t*-test utilizing Levene's test for equality of variances yielded significant results (*p* < 0.01) for the six-month follow up PCa knowledge scores (*M* = −0.12, *SD* = 0.04, *n* = 54). Chi-squared analysis was utilized to examine associations for PCa knowledge among the intervention and control groups at the six-month follow up. The results revealed non-significant results for PCa knowledge among the intervention and control groups at the six-month follow up (*p* > 0.05).

Spearman's Rho correlations were obtained for the following covariates: age, income, level of education, healthcare coverage, PCa screening at the six-month follow up, HCEQ, religion, DSES, AS knowledge, PCa knowledge at the six-month follow up, and PDMS. Table 4 lists the correlations. Age was significantly correlated with religion (*rs* = 0.499, *p* < 0.01). Income was found to be significantly correlated with education (*rs* = 0.535, *p* < 0.01), healthcare coverage (*rs* = .528, *p* < 0.01), and PCa knowledge at the six-month follow up (*rs* = 0.424, *p* < 0.01). A participant's level of education was significantly correlated with PCa knowledge at the six-month follow up (*rs* = 0.450, *p* < 0.01). Prostate cancer screening at the six-month follow up was significantly correlated with religion (*rs* = 0.353, *p* < 0.01). A participant's sense of empowerment indicated by the HCEQ was significantly correlated with his sense of preparation for making a healthcare decision according to the PDMS (*rs* = 0.421, *p* < 0.01). A significant correlation persisted among DSES score and a participant's knowledge of active surveillance (*rs* = 0.377, *p* < 0.01). Furthermore, knowledge of active surveillance was significantly correlated with PCa knowledge (*rs* = 0.497, *p* < 0.01). No other correlations were found to be significant among the covariates.

**Table 4.** Bivariate Correlation Matrix for Age, income, PCa knowledge, AS knowledge, DSE, PDMS, healthcare coverage, PCa post screening, and HCEQ Variables (n = 54).

*Variables*		*1*	*2*	*3*	*4*	*5*	*6*	*7*	*8*	*9*	*10*	*11*
*1.*	*Age*	–										
*2.*	*Income*	−0.017	–									
*3.*	*education*	0.128	0.535**	–								
*4.*	*Health care coverage*	0.064	0.528**	0.183	–							
*5.*	*6 Month prostate cancer screening*	0.256	−0.081	−0.081	0.006	–						
*6.*	*HCEQ*	−0.091	0.193	0.073	0.024	−0.012	–					
*7.*	*Religion*	0.499**	−0.057	−0.051	0.086	0.353**	0.138	–				
*8.*	*DSES*	0.096	0.126	0.148	−0.051	−0.031	0.102	0.020	–			
*9.*	*AS knowledge*	0.229	0.198	0.105	0.212	0.125	0.087	0.021	0.377**	–		
*10.*	*6 Month prostate cancer knowledge*	0.228	0.424**	0.450**	0.148	0.087	0.162	−0.056	0.252	0.497**	–	
*11.*	*PDMS*	−0.169	−0.102	−0.082	−0.089	0.078	0.421**	0.020	0.082	0.106	0.062	–

* *p* < 0.05; ** *p* < 0.01.

## 4. Discussion

We have reported an increase in PCa screening rates among the intervention and control groups at the six-month follow up. At the six-month follow up, 14 participants out of the original 76 reported having received PCa screening. These results are consistent with those of previous studies regarding an increase in PCa screening after an educational intervention [11],[39],[44]. Even though the control groups did not receive the educational intervention, a small number of those participants (21.4%) reported receiving PCa screening. Could the study itself have increased awareness and provided the impetus to seek PCa screening? It is also very likely that given the larger percentage of participants in the control group which did not receive PCa screening, could indicate that the intervention group was more knowledgeable regarding PCa screening. Increasing awareness of PCa among AAs remains an area to focus interventions to educate not only men but also their spouses and partners. Once the seed is planted to engage in health-screening behaviors (e.g., PCa screening), it can be nurtured for growth and completion by the women involved in the lives of AA men.

Women have proven to be a vital motivator for AA men to receive PCa education and screening and engaging in additional health-screening behaviors [45]–[48]. Previous studies regarding predictors for PCa screening indicated marital status as a salient factor [7],[33],[49]. Similarly, the women involved in the lives of AA men have also been a source of social support for AA men to engage in PCa screening [48]. The women that attended the intervention with the men did not participate in the study. However could their presence at the intervention been perceived by their spouse as a display of support? Empirical data have indicated social support as being a predictor and/or associated with AA men receiving PCa screening [50],[51]. It is through an increase in PCa awareness that the health disparity of PCa among AA men can be addressed among the AA population, which is known for decreased preventative health behaviors [33].

Our descriptive results revealed an increase in PCa knowledge and PCa screening. However, PCa knowledge among the groups was not statistically significant at the six-month follow up. The length of time between the baseline questionnaires and/or the intervention may have attributed to the lack of significant results for PCa knowledge among participants. Despite a lack of statistically significant results for PCa knowledge among participants at the six-month follow up, there was an increase in receiving PCa screening. An increase in PCa screening provides hope for increasing awareness and communication regarding PCa. Increasing communication pertaining to PCa within the AA community can provide a foundation for increasing knowledge of PCa and PCa screening which may lead to healthier outcomes for those diagnosed with PCa.

Age has been known to be a predictor of PCa screening in previous studies, which indicates support of the significant findings for age in this study [52]. We found a moderate to strong relation among the correlational analysis between age and religion. An increase in participant age was associated with an increase in level of religiosity. However, a comparison of religiosity scores between the intervention and control groups did not reveal a substantial difference, *M* = 88 (intervention) and *M* = 85 (control). Religion has been shown to be a vital facet in the lives of AAs in the United States [53]. As individuals grow older, they may feel a desire to increase their religious practices or beliefs in preparation for what they believe will be another type of life after their physical death. Religion and churches have also been shown to be influential in promoting healthy lifestyles and practices [39]. Empirical literature reveals religious involvement may improve an individual's well-being through managing health behaviors, which can decrease the possibility of disease [54]. Health information is frequently disseminated among the AA population through the church [39]. Literature indicates health conditions and outcomes can be improved through collaborations with Black churches, which utilize health promoting interventions [55]. Previous studies cited the church as a possible venue for bringing awareness to the concept of informed decision making for PCa screening [56]. Empirical studies identified a link between religion and choices that pertain to one's health [57]. For example, a church based program for PCa screening among AA men found that the church pastors may influence increases in PCa knowledge and rates of PCa screening [56].

Income exhibited a moderate to strong relation among the covariates of “education”, “healthcare coverage”, and “PCa knowledge”. The majority of participants were characterized by a high socioeconomic status (SES), as indicated by an income of at least $50,000. A higher level of income may provide increased access to higher education and healthcare coverage in comparison to individuals with lower levels of income. When individuals possess low levels of education, they are less likely to engage in PCa screening [32]. Our study revealed a strong to moderate level of association among education and PCa knowledge. Education appeared to be important to our participants, only 6% of our cohort did not complete high school. We would be remiss not to acknowledge education as a motivator for the acquisition of PCa knowledge. A correlation study regarding PSA testing among a multiethnic cohort found an association among concern over PCa and undergoing PCa screening [33]. Concerted efforts must be made to educate AA men about their risk factors and to open the lines of communication on the topic, which is deemed sensitive by many individuals. Realizing the vast influence education has on one's well-being, it is not difficult to imagine how it is intertwined with the factor of income. Previous studies have demonstrated a significant association among PSA testing and education and income [58]–[60]. As indicated in previous studies and the current study, the association among income and health insurance remains as an impediment or a facilitator for the health disparity of PCa among AA men. Often times, the lack of health insurance is a barrier for AA men to receive healthcare that is either preventative or simply routine care for an ailment [61].

### Limitations

The limitations of this study included its small sample size, which did not allow us to generalize our findings. The small sample size and attrition rate of 29% is indicative of the lack of retention of AA men in research studies. This apprehension is known to be rooted in the previous atrocities suffered by AA men involved in research such as those of the Tuskegee experiment [62],[63]. Additionally, a participant's PCa screening status was obtained via self-reporting, which may have been impacted by memory recall. Medical documentation of the date of a participant's last PCa screening was not required for participation in the study.

Despite the practicality of a quasi-experimental design for a community research study, the design is also a limitation. We also chose to focus on the relations among PCa knowledge and screening, AS knowledge, psychosocial factors in an effort to learn more about factors which may be linked to increasing PCa awareness and screening. However, our focus on the relations of those covariates as opposed to examining differences maybe also be viewed as a limitation. The lack of randomization for participant allocation in the intervention and control groups was a limitation and a possible source of bias. Most of the participants in the intervention group were from churches, which may have attributed to an increase in PCa screening and the increased fraction of participants who were married. The churches that agreed to participate in the study desired the educational intervention, which resulted in a skewed number of churches involved in the intervention arm of the study. The increased number of churches may also lead to selection bias. Future studies will include randomization of study sites to decrease the possibility of skewed participation of community sites. African American men who indicated that their last PCa screening occurred over one year ago were included in the study. However, the inclusion of men who had previously been screened may have confounded the results of the study. The men who had previously been screened over one year ago, as indicated at the baseline, were included in the study due to the stigma and sensitive nature of PCa screening. There was obviously some element that was preventing these men—who were at a high risk for developing PCa—from undergoing screening despite their previous screening. That element could have been feelings of shame, a lack of support, or uncertainty regarding the benefit of the PCa screening. Therefore, we would have been remiss not to include the fraction of men who indicated having undergone PCa screening at least one year ago. Lastly, there was a missed opportunity to obtain qualitative data from the spouses or partners of the participants in the intervention group. Future research will encompass a qualitative aspect to obtain data from the family or significant others which are involved the well-being of AA men,

## 5. Conclusion

The current study adds to the literature on PCa education, screening, and decision making among AA men in a longitudinal from. Our results revealed an increase in AA men's knowledge and rates of PCa screening based on descriptive data. The findings demonstrated a retention of knowledge and the desire to engage in PCa screening after a six-month follow up. Therefore, in a group that is known to be less likely to engage in health-promoting behaviors or seek preventative care, these men contradicted what has been known. Research must continue to evaluate the effectiveness of PCa educational interventions in an effort to reduce the health disparity of PCa among AA men. Likewise, there must be an increase in communication regarding the high risk of PCa among AA/Black individuals. Until discussions on PCa can be conducted without feelings of shame or embarrassment, men will continue to miss opportunities to seek PCa screening. Discussions on PCa must become more common and as routine if one was discussing his or her high blood pressure. While more research must be forthcoming to evaluate the effectiveness of recommending against PCa screening, there remains a need for continued efforts to educate AA men about their risk of PCa. Through an increase in awareness of the risk of PCa in AA men, a concerted effort can be made to decrease the health disparity of PCa among AA men.
